# Experience in other segments should shorten studies using Look-Locker and high-resolution T2 images in the study of focal lung lesions

**DOI:** 10.1590/0100-3984.2024.57.e8-en

**Published:** 2024-10-14

**Authors:** Marcelo Souto Nacif

**Affiliations:** 1 Adjunct Professor in the Department of Radiology at Universidade Federal Fluminense (UFF), Niterói, RJ, Brazil. Email: msnacif@gmail.com.

The use of magnetic resonance imaging for quantitative assessment is increasingly relevant and has positive impacts on clinical management. The search for nonqualitative tissue characterization and the possibility of differentiating between pathological tissue and normal tissue has been of great value in various organs. The capacity to report on the existence of cell proliferation, the expansion of the interstitial space, or even the presence of edema or inflammation has led to the rapid development of quantitative sequences for implementation in the clinical setting.

The possibility of noninvasive methods producing diagnoses that are very close to those made by traditional methods, without, in some cases, the need to perform biopsy, is of extreme importance in our field. This practice is already very well established in the study of the prostate and breast^(1,2)^. Personalized medicine and the strong focus on genetic studies to predict who is most likely to have cancer or even to treat each type of tumor more accurately will, without a shadow of a doubt, provide better prognoses for patients and increase the survival of the population^(3,4)^.

A prospective observational study, authored by Wada et al.^(5)^ and recently published in Radiologia Brasileira, describes, for the first time, the use of T1 relaxometry and high-resolution T2 techniques for the evaluation of lung lesions, in a sample of 39 individuals. Although accuracy is still difficult to extrapolate, because of the heterogeneity of the lesions and the small number of individuals studied, we agree that both techniques have great potential, as demonstrated by the authors. High-resolution T2 sequences have shown excellent performance in evaluating the morphological characteristics of lesions and especially for demonstrating pseudocavities and pleural tags^(5)^.

Many studies in other fields, such as that of cardiomyopathy, have already demonstrated the importance of the T1 relaxometry technique and have validated it through endomyocardial biopsies^(6)^, Bloch simulations^(7)^, correlation with other techniques such as modified Look-Locker Inversion recovery (MOLLI) sequences^(8)^, and application in large multicenter studies^(9)^. The use of Look-Locker T1 relaxometry is widely accepted in cardiology and is now being replaced by MOLLI precisely to avoid movement artifacts and incorrect pixel fitting in the sequence analysis^(7,8)^.

The postprocessing of Look-Locker images can actually influence the results. Having a description of the standard and how the analysis was done is always important for the correct use of such techniques^(10)^. The same problems related to acquisition time and respiratory movement, in addition to postprocessing, may be present in the evaluation of T2 sequences, which truly represent a great leap forward in the morphological evaluation of lesions, especially with the most modern sequences^(11)^.

We believe that studies such as that conducted by Wada et al.^(5)^ could promote greater discussion on the subject and the search for more consistent alternatives through the use of sequences with less variability and greater tissue characterization capacity. Such alternatives might allow the noninvasive differentiation between benign and malignant tissue.
